# Amoxicillin modulates gut microbiota to improve short-term high-fat diet induced pathophysiology in mice

**DOI:** 10.1186/s13099-022-00513-0

**Published:** 2022-10-13

**Authors:** Suresh Kumar, V. Samuel Raj, Ayaan Ahmad, Vikram Saini

**Affiliations:** 1 National Institute of Biologicals, Ministry of Health & Family Welfare, Govt. of India, Noida, 201309 India; 2 Center for Drug, Design, Discovery and Development (C4D), SRM University, Delhi-NCR, 131029 Sonepat, Haryana India; 3 Laboratory of Infection Biology and Translational Research, Department of Biotechnology, All India Institute of Medical Sciences, New Delhi, India; 4 Biosafety Laboratory-3, Centralized Core Research Facility (CCRF), All India Institute of Medical Sciences (AIIMS), New Delhi, India

**Keywords:** High-fat diet, Gut microbiota, Metabolic syndrome, Pathogens, Amoxicillin, Immune cells

## Abstract

**Background:**

A high-fat diet (HFD) induced perturbation of gut microbiota is a major contributory factor to promote the pathophysiology of HFD-associated metabolic syndrome. The HFD could also increase the susceptibility to the microbial infections warranting the use of antibiotics which are independently capable of impacting both gut microbiota and metabolic syndrome. Further, the usage of antibiotics in individuals consuming HFD can impact mitochondrial function that can be associated with an elevated risk of chronic conditions like inflammatory bowel disease (IBD). Despite this high propensity  to infections in individuals on HFD, the link between duration of HFD and antibiotic treatment, and its impact on diversity of the gut microbiome and features of metabolic syndrome is not well established. In this study, we have addressed these knowledge gaps by examining how the gut microbiota profile changes in HFD-fed mice receiving antibiotic intervention in the form of amoxicillin. We also determine whether antibiotic treatment in HFD-fed mice may adversely impact the ability of immune cells to clear microbial infections.

**Methods and Results:**

We have subjected mice to HFD and chow diet (CD) for 3 weeks, and a subset of these mice on both diets received antibiotic intervention in the form of amoxicillin in the 3rd week. Body weight and food intake were recorded for 3 weeks. After 21 days, all animals were weighted and sacrificed. Subsequently, these animals were evaluated for basic haemato-biochemical and histopathological attributes. We used 16S rRNA sequencing followed by bioinformatics analysis to determine changes in gut microbiota in these mice. We observed that a HFD, even for a short-duration, could successfully induce the partial pathophysiology typical of a metabolic syndrome, and substantially modulated the gut microbiota in mice. The short course of amoxicillin treatment to HFD-fed mice resulted in beneficial effects by significantly reducing fasting blood glucose and skewing the number of thrombocytes towards a normal range. Remarkably, we observed a significant remodelling of gut microbiota in amoxicillin-treated HFD-fed mice. Importantly, some gut microbes associated with improved insulin sensitivity and recovery from metabolic syndrome only appeared in amoxicillin-treated HFD-fed mice reinforcing the beneficial effects of antibiotic treatment in the HFD-associated metabolic syndrome. Moreover, we also observed the presence of gut-microbiota unique to amoxicillin-treated HFD-fed mice that are also known to improve the pathophysiology associated with metabolic syndrome. However, both CD-fed as well as HFD-fed mice receiving antibiotics showed an increase in intestinal pathogens as is typically observed for antibiotic treatment. Importantly though, infection studies with *S. aureus* and *A. baumannii*, revealed that macrophages isolated from amoxicillin-treated HFD-fed mice are comparable to those isolated from mice receiving only HFD or CD in terms of susceptibility, and progression of microbial infection.  This finding  clearly indicated that amoxicillin treatment does not introduce any additional deficits in the ability of macrophages to combat microbial infections.

**Conclusions:**

Our results showed that amoxicillin treatment in HFD-fed mice exert a beneficial influence on the pathophysiological attributes of metabolic syndrome which correlates with a significant remodelling of gut microbiota. A novel observation was the increase in microbes known to improve insulin sensitivity following amoxicillin treatment during short-term intake of HFD. Even though there is a minor increase in gut-resistant intestinal pathogens in amoxicillin-treated groups, there is no adverse impact on macrophages with respect to their susceptibility and ability to control infections. Taken together, this study provides a proof of principle for the exploration of amoxicillin treatment as a potential therapy in the people affected with metabolic syndrome.

## Background

High-fat diet (HFD) consumption can have serious adverse effects on human metabolic and immune health. The HFD sparks chronic low-grade systemic inflammation that can modulate gut microbiota by increasing endotoxins, circulating free fatty acids, and inflammatory mediators, which may impede the homeostasis in many organs [1–3]. Several studies have shown that long-term consumption of a high-fat diet modulates mice’s gut microbiota composition and functionality. Further, a high-fat diet develops the pathophysiology of metabolic disorders by dysbiosis of gut microbiota [4–7]. Individuals with HFD-associated metabolic syndrome are often susceptible to microbial infections. This may warrant the use of antibiotics that can further alter the microbiota resulting in  dysregulation of host immune homeostasis, poor nutrient digestion, and increased risk to opportunistic pathogens owing to a reduced intestinal colonization resistance [8, 9].

However, very little knowledge is available as to how antibiotic treatment in the backdrop of HFD may impact gut microbiota, haemato-biochemical parameters and histopathological changes in an organism. This knowledge is especially relevant now as the modern diet is often rich in fat, and antibiotics are administered during infections to treat patients. However, how antibiotic treatment potentially impacts resident microbiota and macrophages function to play a promoting or inhibitory role affecting the physiology and health of a person on HFD remains unknown.

In this study, we addressed these gaps by performing experiments wherein we provided HFD to mice for 3-weeks and subjected these mice to amoxicillin treatment during the 3rd week to mimic a typical course of antibiotics. The choice of amoxicillin as the preferred antibiotic in this study was based on it being the most common antibiotic used in primary healthcare settings [10]. We first showed that short-term intake of HFD for 3-weeks induces partial physiopathology of metabolic syndrome by disruption of homeostasis of gut microbiota. By using 16S rRNA high-throughput sequencing, we examined the changes in the caecal microbiota and discussed how changes in microbiota composition may promote or suppress the features associated with metabolic pathophysiology in the presence of amoxicillin. We also scored for the changes in haemato-biochemical profile, and histo-pathological analysis on vital organs, namely the heart, liver and kidney in these mice. Finally, we also demonstrated that amoxicillin treatment does not adversely affect the functional capacity of macrophages of clearing infection in the HFD-fed mice. Collectively, our study provides strong evidence to explore amoxicillin-mediated modulation of gut-microbiota as a potential intervention for improving the health of people affected with metabolic syndrome.

## Results

### Effects of HFD and amoxicillin treatment in HFD-fed mice on body weight and feed intake

**Fig. 1:**
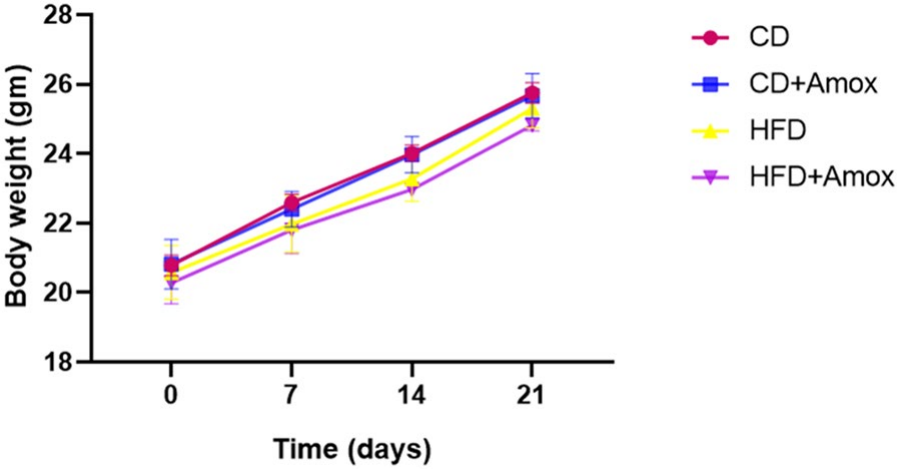
Effect of high-fat diet (3 weeks) and amoxicillin treatment on body weight of mice. As shown by the data, no significant difference in body weight of mice was observed among different groups. Data represent mean ± SD; N = 6 mice/group, One-way ANOVA followed by Bonferroni post-hoc test (* *p* < 0.05, ** *p* < 0.01, *** *p* < 0.001). CD: Standard-chow diet for 3 weeks; CD + Amox: Standard-chow diet for 3 weeks and subjected to amoxicillin treatment in  3rd week; HFD: High-fat diet for 3 weeks; HFD + Amox: High-fat diet for 3 weeks and subjected to amoxicillin treatment in 3rd week

**Fig. 2:**
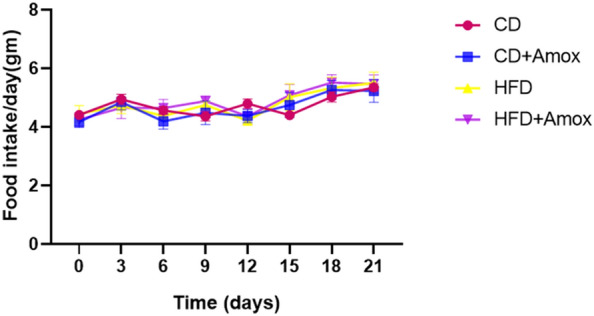
Daily feed intake of mice subjected to high-fat diet and amoxicillin treatment. As shown by the data, no significant difference on daily feed intake of mice was observed among different groups. Data represents mean ± SD; N = 6 mice/group, One-way ANOVA followed by Bonferroni post-hoc test (* *p* < 0.05, ** *p* < 0.01, *** *p* < 0.001). CD: Standard- chow diet for 3 weeks; CD + Amox: Standard- chow diet for 3 weeks and subjected to amoxicillin treatment in  3rd week; HFD: High-fat diet for 3 weeks; HFD + Amox: High-fat diet for 3 weeks and subjected to amoxicillin treatment in 3rd week

As shown in Figure 1, mice fed on HFD  for 3 weeks did not show any significant differences in weight when compared to CD-fed mice. Interestingly, amount of  feed consumed was similar in all groups including those receiving amoxicillin as shown in Figure 2. Likewise, we also did not observe significant weight differences between HFD-fed mice and amoxicillin-treated HFD-fed mice. Also, there were no significant differences in the water intake among different groups.

### Amoxicillin treatment restores the HFD-promoted thrombocytosis

**Fig. 3:**
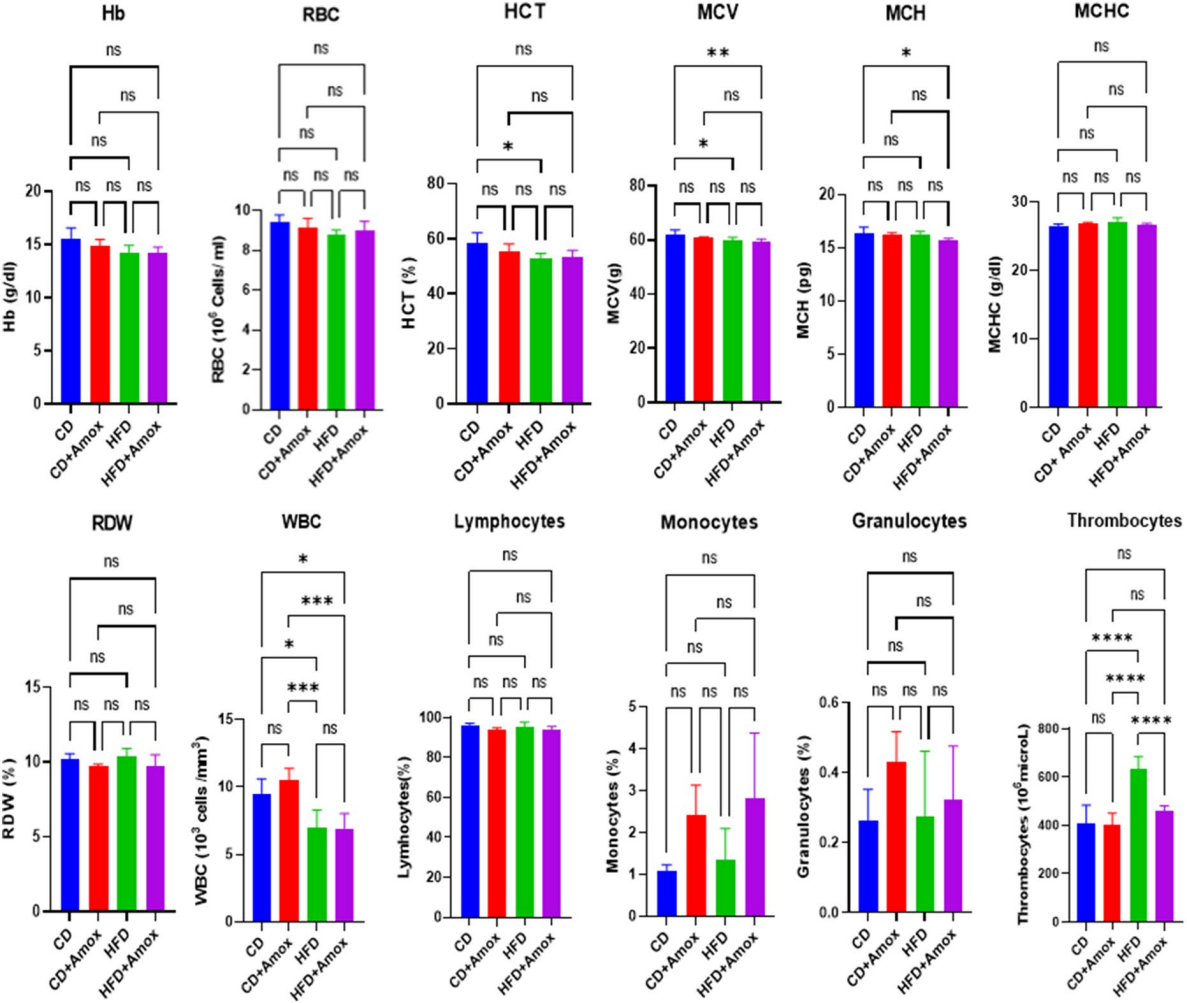
Effect of high-fat diet (3 weeks) and amoxicillin treatment on haematological parameters in mice. HFD treatment for 3 weeks in mice induced the depressive effect on thrombocytes. Amoxicillin treatment in HFD mice in the 3rd week caused restoration of the thrombocytes towards the normal range. Values are mean ± SD for 6 samples in each group. Statistically significance of differences was evaluated by One-way ANOVA followed by Bonferroni post-hoc test (* *p* < 0.05, ** *p* < 0.01, **** *p* < 0.0001). CD: Standard-chow diet for 3 weeks; CD + Amox: Standard-chow diet for 3 weeks and subjected to amoxicillin treatment in 3rd week; HFD: High-fat diet for 3 weeks; HFD + Amox: High-fat diet for 3 weeks and subjected to amoxicillin treatment in 3rd week

We next evaluated the impact of the HFD and amoxicillin on haematological parameters (Figure 3 and Table 1). Compared to CD, in HFD, we observed a slight decline in the value of haemoglobin (Hb), red blood cells (RBC), mean corpuscular haemoglobin (MCH), and a significant reduction in haematocrit (HCT, *p* = 0.022), mean corpuscular volume (MCV, *p* = 0.02189) and white blood cells (WBCs, *p* = 0.0128). Moreover, HFD-treated mice significantly increased thrombocytes (*p* < 0.0001) compared to the CD group. On the other hand, amoxicillin-treated HFD-fed mice could decrease the thrombocytes (*p* < 0.0001) significantly towards normal range without any significant alterations in HCT and MCV values. Nonetheless, we did not observe any significant change in haematological parameters in the mice belonging to CD + Amox group or CD group alone. Based on this data, it is clear that amoxicillin treatment in CD-fed mice did not affect thrombocyte value while it exerted a positive effect on restoring their number towards normalcy in the HFD-fed mice.

**Table 1:** Effect of diet including HFD used in this study on various haematological parameters

Parameters	Groups				Significance (*p* value*)*		
	CD	CD + A	HFD	HFD + A	CD vs CD + A	CD vs HFD	HFD vs HFD + A
Hb (g/dl)	15.46 ± 1.1	14.83 ± 0.6	14.26 ± 0.6	14.16 ± 0.5	NS	NS	NS
RBC (10^6^ cells/ml)	9.40 ± 0.3	9.1 ± 0.4	8.77 ± 0.24	8.98 ± 0.4	NS	NS	NS
HCT (%)	58.23 ± 3.8	55.23 ± 2.8	52.56 ± 2.1	53.46 ± 2.2	NS	0.0226	NS
MCV (g)	61.9 ± 1.8	60.76 ± 0.3	60.0 ± 1.04	59.6 ± 0.7	NS	0.0218	NS
MCH (pg)	16.36 ± 0.5	16.26 ± 0.1	16.2 ± 0.3	15.7 ± 0.2	NS	NS	NS
MCHC(g/dl)	26.5 ± 0.2	26.83 ± 0.2	27.1 ± 0.6	26.6 ± 0.2	NS	NS	NS
RDW (%)	10.2 ± 0.34	9.72 ± 0.11	10.3 ± 0.57	9.7 ± 0.78	NS	NS	NS
WBC (10^6^ cells/ml)	9.41 ± 1.1	10.52 ± 0.8	6.95 ± 1.3	6.92 ± 1.1	NS	0.0128	NS
Lymphocytes (10^6^ cells/ml)	96.3 ± 0.7	93.6 ± 1.3	95.0 ± 2.7	93.6 ± 1.9	NS	NS	NS
Monocytes (%)	1.06 ± 0.1	2.4 ± 0.7	1.33 ± 0.7	2.8 ± 1.5	NS	NS	NS
Granulocytes (%)	0.26 ± 0.08	0.43 ± 0.08	0.27 ± 0.1	0.32 ± 0.1	NS	NS	NS
Thrombocytes (10^3^/microL)	407.6 ± 76.2	400.3 ± 50.5	630.6 ± 55.08	459 ± 21.9	NS	< 0.0001	< 0.0001

Values are expressed as the mean ± SD (n = 6). Data were analysed by One-way ANOVA followed by Bonferroni post-hoc test, *p* ≤ 0.05, *NS* = not significant, CD: Standard-chow diet for 3 weeks; CD + A: Standard-chow diet for 3 weeks and subjected to amoxicillin treatment (50 mg/kg body weight) in 3rd week; HFD: High-fat diet for 3 weeks (73% energy from fat); HFD + A: High-fat diet for 3 weeks and subjected to amoxicillin treatment (50 mg/kg body weight) in 3rd week

### Amoxicillin treatment moderates the elevated blood glucose level associated with HFD

**Fig. 4:**
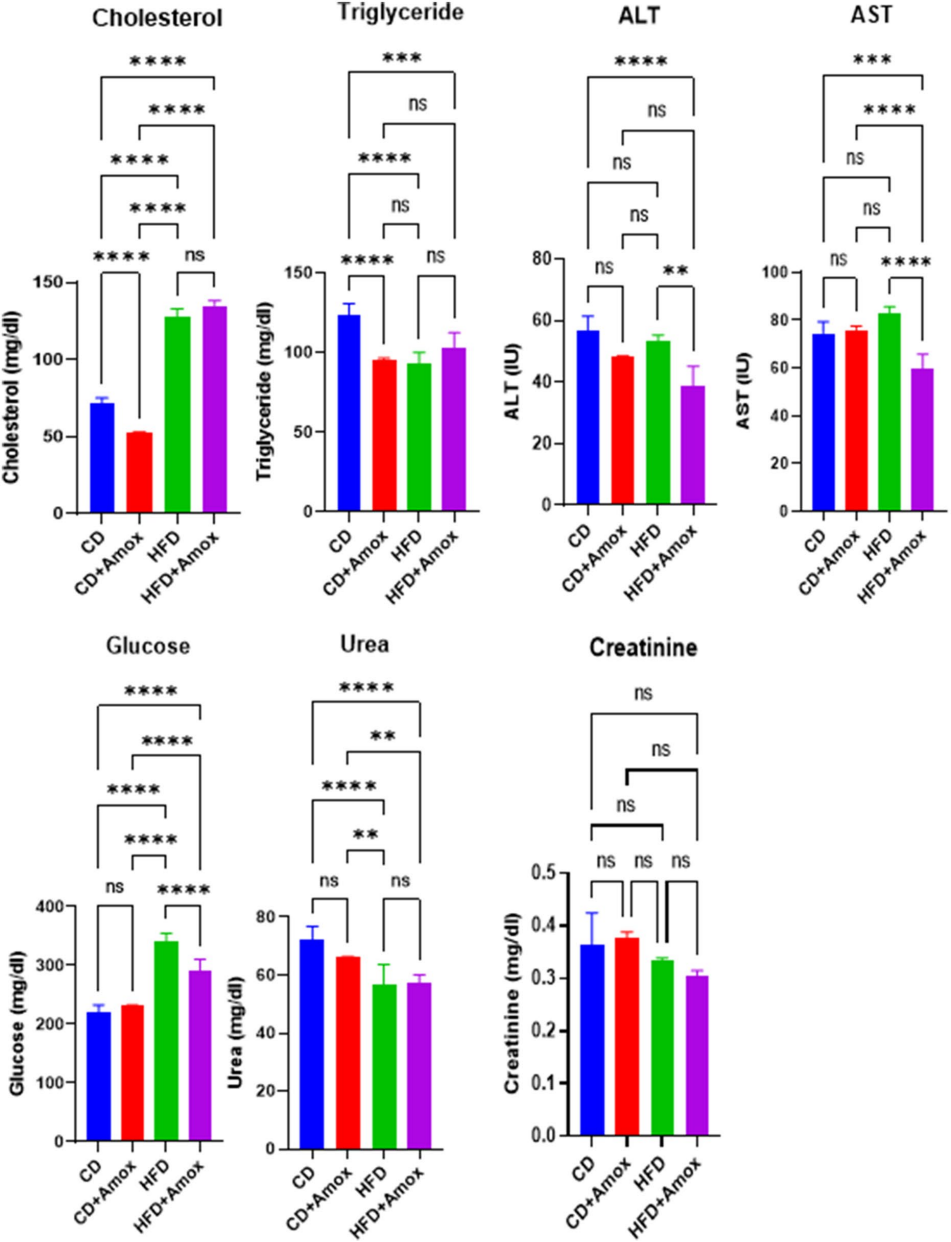
Effect of high-fat diet (3 weeks) and amoxicillin treatment on biochemical parameters in mice. HFD treatment for 3 weeks in mice showed a significant increase in total cholesterol and glucose and a significant decrease in triglycerides. Amoxicillin treatment in HFD mice in the 3rd week caused restoration of the blood glucose level towards the normal range. Values are mean ± SD; N = 6 mice/group. Statistical significance of differences was evaluated by One-way ANOVA followed by Bonferroni post-hoc test (* *p* < 0.05, ** *p* < 0.01, *** *p* < 0.001, **** *p* < 0.0001). CD: Standard-chow diet for 3 weeks; CD + Amox: Standard-chow diet for 3 weeks and subjected to amoxicillin treatment in 3rd week; HFD: High-fat diet for 3 weeks; HFD + Amox: High-fat diet for 3 weeks and subjected to amoxicillin treatment in 3rd week

Analysis of biochemical parameters (Figure 4 and Table 2) show that levels of *Alanine aminotransferase* (ALT), *Aspartate aminotransferase* (AST), and creatinine values are not significantly different in mice receiving standard CD or short-term HFD. However, we did observe a significant increase in total cholesterol and glucose (*p* < 0.0001) levels in the HFD-fed mice *vis a vis* mice fed on normal CD. Further, amoxicillin treatment of HFD- fed mice did not significantly change levels of triglycerides, cholesterol and urea as compared to mice fed only on HFD. Interestingly, amoxicillin treatment significantly reduced glucose levels in HFD-fed mice (*p* < 0.0001). Further, amoxicillin also improved other biochemical parameters of blood and restored levels of liver enzyme AST (*p* < 0.0001) in mice on a HFD. Interestingly, the mice administered standard CD and receiving amoxicillin treatment showed a  significant reduction in the levels of cholesterol, triglycerides and urea as compared to the only CD group. For an inter-group comparison between CD and HFD-fed, we did not observe any significant difference in ALT and creatinine concentration. Thus, our data suggest that amoxicillin treatment  in HFD-fed mice improves levels of blood glucose and AST suggesting a possible beneficial impact on metabolic pathophysiology.

**Table 2:** Effect of diet including HFD used in this study on various biochemical parameters

Parameters	Groups				Significance (*p* value*)*		
	CD	CD + A	HFD	HFD + A	CD vs CD + A	CD vs HFD	HFD vs HFD + A
Cholesterol (mg/dl)	71.49 ± 3.6	52.7 ± 0.3	127.39 ± 5.5	135.08 ± 3.1	< 0.0001	< 0.0001	NS
Triglycerides(mg/dl)	122.99 ± 7.5	95.44 ± 0.9	92.79 ± 7.1	102.41 ± 9.8	< 0.0001	< 0.0001	NS
ALT (IU)	56.80 ± 4.5	48.04 ± 0.5	53.12 ± 2.1	38.82 ± 6.3	NS	NS	0.0015
AST (IU)	74.47 ± 4.8	75.89 ± 1.6	83.08 ± 2.49	59.61 ± 6.07	NS	NS	< 0.0001
Creatinine (mg/dl)	0.36 ± 0.06	0.37 ± 0.01	0.33 ± 0.005	0.30 ± 0.01	NS	NS	NS
Urea (mg/dl)	72.21 ± 4.5	66.15 ± 0.2	59.57 ± 1.9	57.20 ± 2.97	0.0029	< 0.0001	NS
Glucose (mg/dl)	219.80 ± 13.08	231.54 ± 0.89	340.04 ± 14.7	290.77 ± 19.8	NS	< 0.0001	< 0.0001

Values are expressed as the mean ± SD (n = 6). Data were analyzed by One-way ANOVA followed by Bonferroni post-hoc test, *p* ≤ 0.05, *NS=* not significant, CD: Standard-chow diet for 3 weeks; CD + A: Standard-chow diet for 3 weeks and subjected to amoxicillin treatment (50 mg/kg body weight) in 3rd week; HFD: High-fat diet for 3 weeks (73% energy from fat); HFD + A: High-fat diet for 3 weeks and subjected to amoxicillin treatment (50 mg/kg body weight) in 3rd week

### Amoxicillin treatment decreases HFD induced richness and diversity of specific gut bacteria

**Fig. 5:**
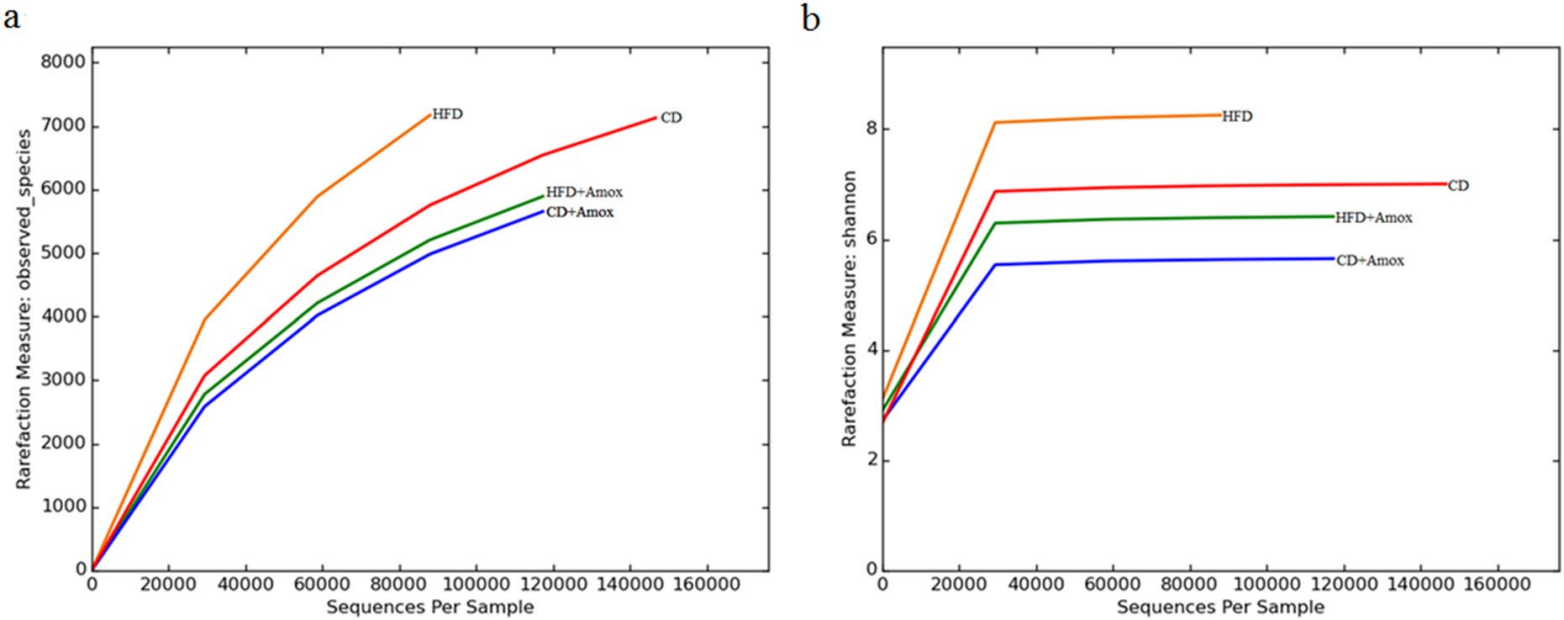
Microbiota diversity in mice subjected to high-fat diet (3 weeks) and amoxicillin treatment. Rarefaction curves (**a),** and Shannon–Wiener curve (**b).** The plateuing observed in Shannon–Wiener curve suggested that the number of OTUs was sufficient to capture the authentic bacterial communities in each sample. CD: Standard-chow diet for 3 weeks; CD + Amox: Standard-chow diet for 3 weeks and subjected to amoxicillin treatment in 3rd week; HFD: High-fat diet for 3 weeks; HFD + Amox: High-fat diet for 3 weeks and subjected to amoxicillin treatment in 3rd week

Considering an improved glucose profile observed in HFD-fed mice treated with amoxicillin, we posited that these metabolic changes may be indicative of the changes in the gut microbiome associated with pathophysiology of metabolic syndrome. For this purpose, we next investigated how amoxicillin treatment with or without HFD can impact the diversity of gut microbiota. Towards this end, we have performed sequencing of 16S rRNA and obtained a total of 11,69,822 high-quality sequences, with an average of 2,92,455 reads per sample. Using a similarity threshold of 97%, we obtained 22,980 as the total number of operating taxonomic units OTUs (Figure 5 and Table 3). The Shannon–Wiener curve, indicating the adequacy of sequencing depth for all samples, achieved a plateau (Figure 5), suggesting that the sequencing has sufficient depth to capture species abundance present in the samples. Next, we determined diversity using Chao1 (richness of species index), Shannon (rare species index), Simpson (common species index) indices, and number of observed species in each group are summarized in Table 3. Both Chao1 and Shannon indices have the highest values in the HFD-fed mice which were significantly reduced following amoxicillin treatment [*p*=0.0002 for Chao1, and *p*<0.0001 for Shannon index]. Similarly, amoxicillin treatment in CD-fed mice significantly reduced value of Chao1 (*p*<0.0002) and Shannon indices (*p*<0.0001).We did not observe a statistically significant difference for the Simpson index among all the four groups. From this data, we concluded that HFD increases the number of specific gut bacteria, while amoxicillin treatment depletes these bacteria.

**Table 3:** Effect of diet on alpha diversity of gut microbiota in mice

Alpha diversity indices	Groups				Significance (*p* value)		
	CD	CD + A	HFD	HFD + A	CD vs CD + A	CD vs HFD	HFD vs HFD + A
OTUs	6468.7 ± 568.6	4871.6 ± 678.5	6543.4 ± 634.1	5104.7 ± 686.5	0.0013	NS	0.0053
Chao1	8682.7 ± 42.69	7152.3 ± 74.4	9354.4 ± 651.4	7500.5 ± 46.09	< 0.0001	0.0229	0.0002
Shannon	6.99 ± 0.0132	5.63 ± 0.020	8.19 ± 0.055	6.39 ± 0.018	< 0.0001	< 0.0001	< 0.0001
Simpson	0.933 ± 1.110	0.912 ± 0.00	0.979 ± 0.00	0.935 ± 0.00	NS	NS	NS

Values are expressed as the mean ± SD (n = 6). Data were analyzed by One-way ANOVA followed by Bonferroni post-hoc test,  *p*≤ 0.05, *NS* =not significant, CD: Standard-chow diet for 3 weeks; CD + A: Standard-chow diet for 3 weeks and subjected to amoxicillin treatment (50 mg/kg body weight) in 3rd week; HFD: High-fat diet for 3 weeks (73% energy from fat); HFD + A: High-fat diet for 3 weeks and subjected to amoxicillin treatment (50 mg/kg body weight) in  3rd week

### Amoxicillin treatment reshapes the gut microbiota associated with improved pathophysiology of metabolic syndrome

**Fig. 6:**
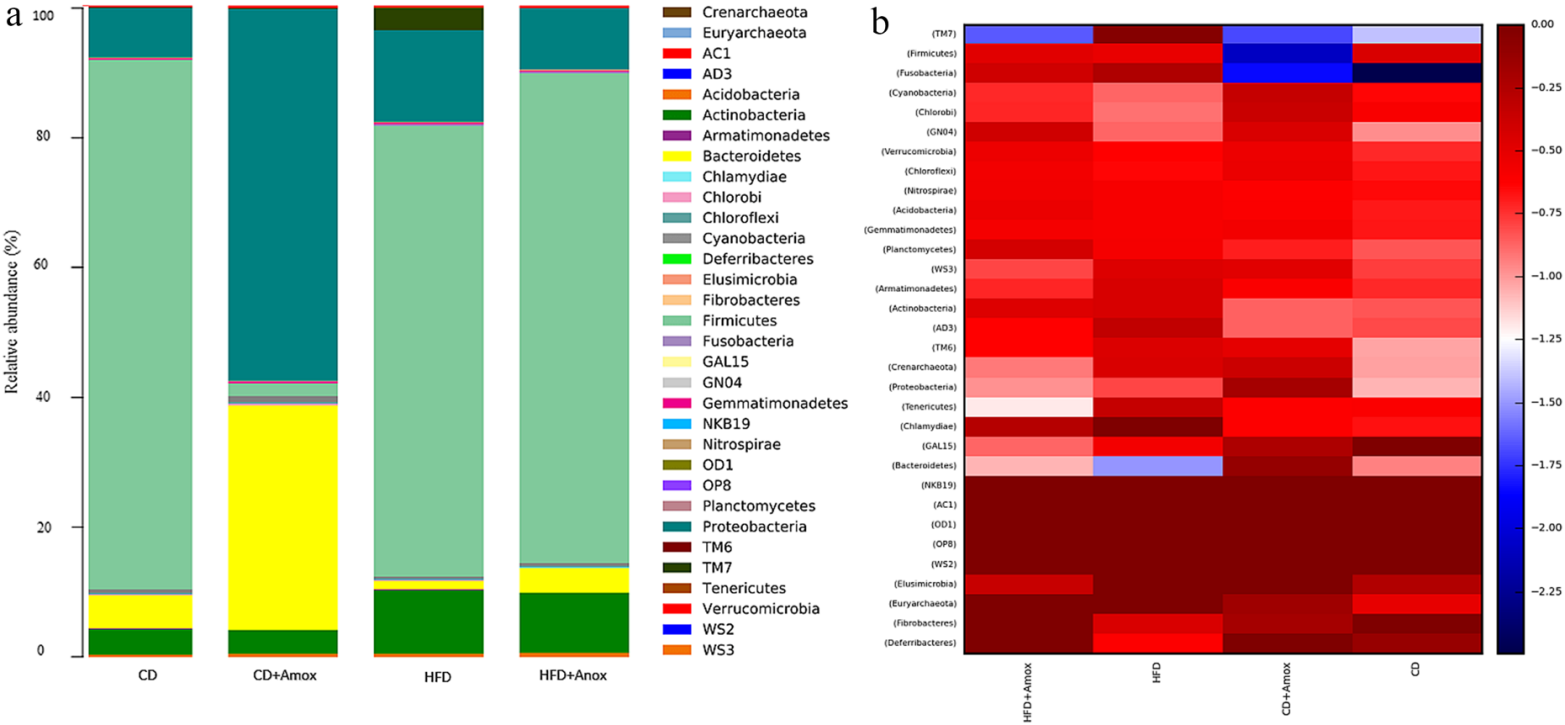
Bar chart (**a)** depicts the effect of high-fat diet (3 weeks) and amoxicillin treatment on the relative abundance of the most represented bacterial taxa at the phylum level for each sample. The x coordinate represents the name of the groups. The y coordinate represents the relative abundance. HFD treatment for 3 weeks in mice showed a significant increase of *Firmicutes* to *Bacteroidetes (F/B)* ratio and *Proteobacteria.* Amoxicillin treatment in HFD mice in the 3rd week caused a significant decrease in the *Firmicutes* to *Bacteroidetes (F/B)* ratio and *Proteobacteria*. The analysis of color heat map (**b)** illustrates the abundance of top 32 mean bacterial community taxa assigned to phyla. The color intensity in each sample is normalized to represent its relative ratio in the four groups. The color scale of dark red and dark blue represents the higher and lower relative abundances of bacterial communities, respectively. CD: Standard- chow diet for 3 weeks, CD + Amox: Standard- chow diet for 3 weeks and subjected to amoxicillin treatment in 3rd  week; HFD: High-fat diet for 3 weeks; HFD + Amox: High-fat diet for 3 weeks and subjected to amoxicillin treatment in 3rd  week

After determining the richness and diversity associated with various treatment groups, we next determined and analyzed the relative abundance of the microbiota at the phylum level (Figure 6 and Table 4). Our analysis revealed that consumption of  HFD in mice caused a significant increase in the *Firmicutes* to *Bacteroidetes* ratio (*F/B* ratio) compared to the CD group (15.46 ± 0.361 to 48.35 ± 0.449, *p* < 0.0001). Interestingly, amoxicillin treatment in HFD mice significantly reduced the *F/B* ratio (48.35 ± 0.449 to 19.30 ± 0.394, *p* < 0.0001) as compared to the group receiving  HFD only. Similarly, amoxicillin treatment of mice fed on CD also showed a significantly decreased *F/B* ratio *(*0.05 ± 0.022, *p* < 0.0001) as compared to the group on CD only. Values are expressed as the mean ± SD.

**Table 4:** Effect of diet on the distribution of gut microbiota in mice at phylum level

Phylum	Relative abundance (%)				Significance (*p* value)		
	CD	CD + A	HFD	HFD + A	CD vs CD + A	CD vs HFD	HFD vs HFD + A
*Firmicutes*	80.4 ± 0.396	1.80 ± 0.132	67.70 ± 0.467	73.70 ± 0.440	< 0.0001	< 0.0001	< 0.0001
*Proteobacteria*	7.50 ± 0.897	56.30 ± 0.496	13.80 ± 0.344	9.20 ± 0.289	< 0.0001	< 0.0001	< 0.0001
*Bacteroidetes*	5.20 ± 0.233	34.00 ± 0.473	1.40 ± 0.117	3.80 ± 0.191	< 0.0001	< 0.0001	< 0.0001
*Actinobacteria*	3.80 ± 0.191	3.60 ± 0.186	9.60 ± 0.294	9.10 ± 0.287	NS	< 0.0001	0.0143
*Verrucomicrobia*	0.20 ± 0.044	0.40 ± 0.063	0.30 ± 0.054	0.30 ± 0.054	< 0.0001	NS	NS
*TM7*	0.10 ± 0.031	0.10 ± 0.031	3.30 ± 0.178	0.10 ± 0.031	NS	< 0.0001	< 0.0001
*Acidobacteria*	0.30 ± 0.054	0.50 ± 0.070	0.60 ± 0.077	0.60 ± 0.077	0.0264	< 0.0001	NS
*Cyanobacteria*	0.50 ± 0.070	0.90 ± 0.094	0.30 ± 0.054	0.40 ± 0.063	< 0.0001	0.0002	0.0435
*F/B* ratio	15.46 ± 0.361	0.05 ± 0.022	48.35 ± 0.449	19.30 ± 0.394	< 0.0001	< 0.0001	< 0.0001

Values are expressed as the mean ± SD (n = 6). Data were analyzed by One-way ANOVA followed by Bonferroni post-hoc test, *p* ≤ 0.05, *NS=* not significant, CD: standard- chow diet for 3 weeks; CD + A: Standard- chow diet for 3 weeks and subjected to amoxicillin treatment (50 mg/kg body weight) in 3rd  week; HFD: high-fat diet for 3 weeks (73% energy from fat); HFD + A: High-fat diet for 3 weeks and subjected to amoxicillin treatment (50 mg/kg body weight) in 3rd week

We further observed that the relative abundance of *Proteobacteria* was significantly higher in HFD-fed mice compared to CD-fed mice (*p* < 0.0001). However, amoxicillin treatment in HFD-fed mice resulted in a significant decrease of *Proteobacteria* compared to the HFD group. In contrast, amoxicillin- treated CD group has a significantly increased relative abundance of *Proteobacteria* compared to the CD group (*p* < 0.0001). A balance of the *F/B* ratio is a widely accepted indicator of  health that influences gut homeostasis. Various research studies  have shown that the increased *F/B* ratio is associated with obesity while the decrease in this ratio is connected to dysbiosis [11–13]. Further, *Proteobacteria* is considered the most variable species that contribute to dysbiosis, while body leanness is positively correlated with *Bacteroidetes* [11, 14]**.**

**Fig. 7:**
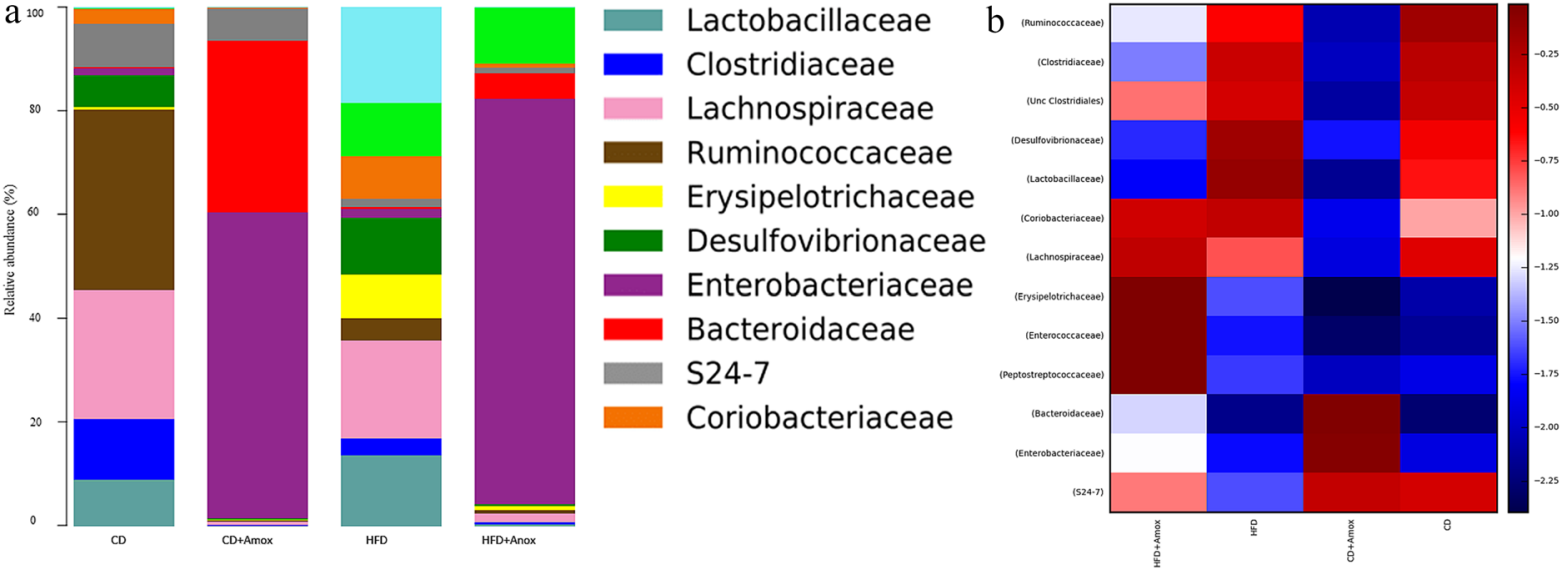
Bar chart (**a**) depicting the effect of high-fat diet (3 weeks) and amoxicillin treatment on the relative abundance of the most represented bacterial taxa at the family level for each sample. HFD treatment for 3 weeks in mice showed a significant decrease of *Lachnospiraceae* and *S24-7.* Amoxicillin treatment in HFD mice in 3rd week caused a significant increase in *Lachnospiraceae* and *S24-7.* The analysis of the color heat map (**b**) illustrates the mean abuandances of top 13 bacterial community taxa assigned to family. The color intensity in each sample is normalized to represent its relative ratio in the four groups. The color scale of dark red and dark blue represents the higher and lower relative abundances of bacterial communities, respectively. CD: Standard- chow diet for 3 weeks; CD + Amox: Standard- chow diet for 3 weeks and subjected to amoxicillin treatment in 3rd  week; HFD: High-fat diet for 3 weeks; HFD + Amox: High-fat diet for 3 weeks and subjected to amoxicillin treatment in 3rd week

Taken together, analysis of the data in this study highlighted the presence of 45, 53, 63, and 61 bacterial families in the CD, CD + Amox, HFD and HFD + Amox groups, respectively (Figure 7 and Table 5). Compared to CD group, we observed a significant reduction of *Lachnospiraceae* and *S24-7* family (*p* < 0.0001) in the HFD group. However, amoxicillin treatment in HFD-fed mice caused a significant increase of *Lachnospiraceae* and *S24*-*7* (*p* < 0.0001), while in CD group, amoxicillin treatment led to a significant decrease of *Lachnospiraceae,* and an increase *of S24-7* (*p* < 0.0001) compared to CD group only*. Lachnospiraceae* produces beneficial metabolites for the host such as short-chain fatty acids (SCFAs) especially propionate, and can provide energy for the maintenance of other gut microbes while supporting the development of host epithelial cells [15]*. *On the other hand*,* reduction in *S24-7* is associated with a decrease in butyrate production that may induce metabolic endotoxemia and various other metabolic disorders [4]. Further, *Enterobacteriaceae a*nd *Bacteroidaceae* were significantly higher in the amoxicillin-treated CD group as compared to CD group alone. The *Enterobacteriaceae* and *Bacteroidaceae* are also usually associated with acute infective processes [16]. Likewise, we also observed a significant increase in  *Erysipelotrichaceae* in HFD-fed mice compared to CD-fed mice. *Erysipelotrichaceae* abundance in HFD-fed mice was further amplified following amoxicillin treatment. *Erysipelotrichaceae *flourishing following  broad-spectrum antibiotic treatment has also been observed previously [17]**.**

**Table 5:** Effect of diet on distribution of gut microbiota of mice at family level

Family	Relative abundance (%)				Significance (Adjusted *p* value)		
	CD	CD + A	HFD	HFD + A	CD vs CD + A	CD vs HFD	HFD vs HFD + A
*Enterococcaceae*	0.0 ± 0.000	0.0 ± 0.000	0.1 ± 0.031	3.1 ± 0.173	NS	NS	< 0.0001
*Lactobacillaceae*	4.5 ± 0.207	0.1 ± 0.031	15.2 ± 0.359	0.3 ± 0.054	< 0.0001	< 0.0001	< 0.0001
*Unc Clostridiales*	39.0. ± 0.487	0.0 ± 0.000	32.6 ± 0.468	11.2 ± 0.315	< 0.0001	< 0.0001	< 0.0001
*Clostridiaceae*	5.9 ± 0.235	0.1 ± 0.031	4.9 ± 0.222	0.3 ± 0.054	< 0.0001	< 0.0001	< 0.0001
*Lachnospiraceae*	12.4 ± 0.329	0.4 ± 0.063	5.7 ± 0.231	17.7 ± 0.381	< 0.0001	< 0.0001	< 0.0001
*Peptostreptococcaceae*	0.1 ± 0.031	0.0 ± 0.000	0.1 ± 0.031	4.7 ± 0.211	< 0.0001	NS	< 0.0001
*Ruminococcaceae*	17.4 ± 0.379	0.2 ± 0.044	6.3 ± 0.242	1.4 ± 0.117	< 0.0001	< 0.0001	< 0.0001
*Erysipelotrichaceae*	0.3 ± 0.054	0.1 ± 0.031	0.8 ± 0.089	33.5 ± 0.471	< 0.0001	< 0.0215	< 0.0001
*Desulfovibrionaceae*	3.0 ± 0.170	0.2 ± 0.044	7.5 ± 0.263	0.2 ± 0.141	< 0.0001	< 0.0001	< 0.0001
*Enterobacteriaceae*	0.7 ± 0.083	47.3 ± 0.499	0.9 ± 0.094	3.3 ± 0.178	< 0.0001	0.0442	< 0.0001
*Bacteroidaceae*	0.2 ± 0.044	26.7 ± 0.442	0.2 ± 0.044	1.4 ± 0.117	< 0.0001	NS	< 0.0001
*S24-7*	4.1 ± 0.198	4.9 ± 0.215	0.3 ± 0.054	1.4 ± 0.117	< 0.0001	< 0.0001	< 0.0001
*Coriobacteriaceae*	1.4 ± 0.117	0.2 ± 0.044	6.7 ± 0.250	5.7 ± 0.231	< 0.0001	< 0.0001	< 0.0001

Values are expressed as the mean ± SD (n = 6). Data were analyzed by One-way ANOVA followed by Bonferroni post-hoc test, *p* ≤ 0.05, *NS=* not significant, CD: Standard- chow diet for 3 weeks; CD + A: Standard- chow diet for 3 weeks and subjected to amoxicillin treatment (50 mg/kg body weight) in 3rd  week; HFD: high-fat diet for 3 weeks (73% energy from fat); HFD + A: High-fat diet for 3 weeks and subjected to amoxicillin treatment (50 mg/kg body weight) in 3rd week. Unc=Unculturable

**Fig. 8:**
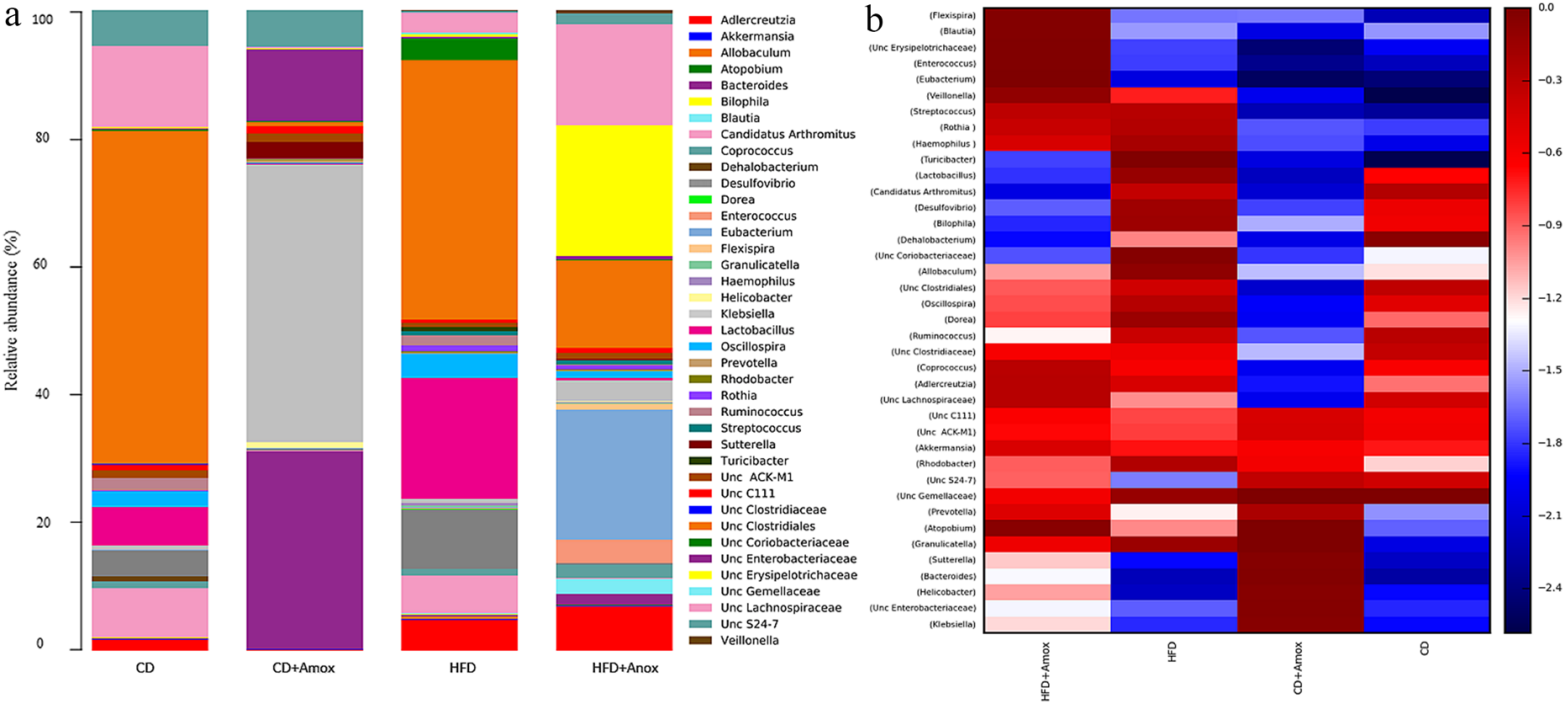
Bar chart (**a)** depicting effect of high-fat diet (3 weeks) and amoxicillin treatment on the relative abundance of the most represented bacterial taxa at the genus level for each sample. Amoxicillin treatment in the 3rd week in the HFD group showed a significant rise in the relative abundance of *Blautia, Coprococcus, Bacteroides*, *Akkermansia* and a decreased in *Granulicatella, Lactobacillus, Streptococcus, Turicibacter, Desulfovibrio, Oscillospira*, and *Allobaculum.* The analysis of the color heat map (**b)** illustrates the mean abundances of top 39 bacterial community taxa assigned to genera. The color intensity in each sample is normalized to represent its relative ratio in the four groups. The color scale of dark red and dark blue represents the higher and lower relative abundances of bacterial communities, respectively. CD: Standard—chow diet for 3 weeks; CD + Amox: Standard—chow diet for 3 weeks and subjected to amoxicillin treatment in 3rd  week; HFD: High-fat diet for 3 weeks; HFD + Amox: High-fat diet for 3 weeks and subjected to amoxicillin treatment in 3rd week

At the genus level, a total of 21, 26, 38, and 37 genera (Figure 8 and Table 6) were observed in CD, CD + Amox, HFD, and HFD + Amox groups, respectively. Further, the relative abundance of microbiota at the species level is shown in Table 7. Data analysis revealed a significant increase in the relative abundance of *Lactobacillus*, *Turicibacter*, *Desulfovibrio*, *Oscillospira*, *Allobaculum,* *Streptococcus*, *Granulicatella*, and *Rothia* (*p* < 0.0001) in HFD-fed mice as compared to CD-fed mice (Table 6). Most of the *Lactobacillus*  strains nourish gut health by secreting anti-microbial substances that stop pathogens from colonizing the gut [18]. HFD-fed mice had a significant reduction in *Lactobacillus reuteri* (*p* < 0.0001) that is helpful in maintaining the integrity of the gut epithelial barrier and prevents the growth of pathogens in the intestine by secreting anti-microbial substances such as *reuteri* [18, 19].

**Table 6:** Effect of diet on mice gut microbiota at genus level

Genus	Relative abundance at genus level (%)				Significance (adjusted *p* value)		
	CD	CD + A	HFD	HFD + A	CD vs CD + A	CD vs HFD	HFD vs HFD + A
*Unc Gemellaceae*	0.0 ± 0.000	0.0 ± 0.000	0.2 ± 0.044	0.01 ± 0.009	NS	< 0.0001	< 0.0001
*Granulicatella*	0.0 ± 0.000	0.0 ± 0.000	0.2 ± 0.044	0.1 ± 0.031	NS	< 0.0001	< 0.0001
*Enterococcus*	0.0 ± 0.000	0.0 ± 0.000	0.1 ± 0.031	3.0 ± 0.170	NS	NS	< 0.0001
*Lactobacillus*	4.5 ± 0.207	0.1 ± 0.031	15.2 ± 0.359	0.3 ± 0.054	< 0.0001	< 0.0001	< 0.0001
*Streptococcus*	0.0 ± 0.000	0.0 ± 0.000	0.6 ± 0.077	0.5 ± 0.070	NS	< 0.0001	0.0341
*Turicibacter*	0.0 ± 0.000	0.0 ± 0.000	0.5 ± 0.070	0.0 ± 0.000	NS	< 0.0001	< 0.0001
*Unc Clostridiales*	39.1 ± 0.489	0.6 ± 0.077	32.7 ± 0.469	11.3 ± 0.316	< 0.0001	< 0.0001	< 0.0001
*Unc Clostridiaceae*	0.0 ± 0.000	0.0 ± 0.000	0.0 ± 0.000	0.2 ± 0.044	NS	NS	< 0.0001
*Candidatus Arthromitus*	5.7 ± 0.232	0.1 ± 0.031	4.8 ± 0.213	0.1 ± 0.031	< 0.0001	< 0.0001	< 0.0001
*Dehalobacterium*	0.6 ± 0.077	0.0 ± 0.000	0.1 ± 0.031	0.0 ± 0.000	< 0.0001	< 0.0001	0.0077
*Unc Lachnospiraceae*	9.5 ± 0.293	0.3 ± 0.054	2.5 ± 0.156	12.8 ± 0.334	< 0.0001	< 0.0001	< 0.0001
*Blautia*	0.1 ± 0.031	0.0 ± 0.000	0.1 ± 0.031	2.0 ± 0.140	< 0.0001	NS	< 0.0001
*Coprococcus*	0.8 ± 0.089	0.0 ± 0.000	0.8 ± 0.089	1.6 ± 0.239	< 0.0001	NS	< 0.0001
*Dorea*	0.0 ± 0.000	0.0 ± 0.000	0.2 ± 0.044	0.0 ± 0.000	NS	< 0.0001	< 0.0001
*Oscillospira*	1.8 ± 0.132	0.1 ± 0.031	3.1 ± 0.173	0.8 ± 0.089	< 0.0001	< 0.0001	< 0.0001
*Ruminococcus*	1.9 ± 0.136	0.0 ± 0.000	0.6 ± 0.077	0.2 ± 0.044	< 0.0001	< 0.0001	< 0.0001
*Veillonella*	0.0 ± 0.000	0.0 ± 0.000	0.1 ± 0.031	0.4 ± 0.063	NS	0.0020	< 0.0001
*Unc Erysipelotrichaceae*	0.2 ± 0.044	0.1 ± 0.031	0.3 ± 0.054	16.6 ± 0.372	0.0019	NS	< 0.0001
*Allobaculum*	0.0 ± 0.000	0.0 ± 0.000	0.4 ± 0.063	0.0 ± 0.000	NS	< 0.0001	< 0.0001
*Eubacterium*	0.1 ± 0.031	0.1 ± 0.031	0.2 ± 0.044	16.6 ± 0.372	NS	NS	< 0.0001
*Rhodobacter*	0.0 ± 0.000	0.1 ± 0.031	0.3 ± 0.054	0.1 ± 0.031	0.0019	< 0.0001	< 0.0001
*Sutterella*	0.0 ± 0.00	2.2 ± 0.146	0.0 ± 0.000	0.2 ± 0.044	< 0.0001	NS	< 0.0001
*Bilophila*	0.0 + 0.000	0.0 ± 0.000	0.1 ± 0.031	0.0 ± 0.00	NS	< 0.0001	< 0.0001
*Desulfovibrio*	3.0 ± 0.170	0.2 ± 0.044	7.4 ± 0.261	0.2 ± 0.044	< 0.0001	< 0.0001	< 0.0001
*Flexispira*	0.0 ± 000	0.0 ± 0.000	0.0 ± 0.000	0.7 ± .083	NS	NS	< 0.0001
*Helicobacter*	0.0 ± 000	0.8 ± 0.089	0.0 ± 0.000	0.1 ± 0.031	< 0.0001	NS	< 0.0001
*Unc Enterobacteriaceae*	0.2 ± 0.044	9.7 ± 0.295	0.2 ± 0.044	0.6 ± 0.077	< 0.0001	NS	< 0.0001
*Klebsiella*	0.5 ± 0.070	37.5 ± 0.484	0.6 ± 0.077	2.7 ± 0.162	< 0.0001	NS	< 0.0001
*Haemophilus*	0.0 ± 000	0.0 ± 000	0.3 ± 0.054	0.1 ± 0.031	NS	< 0.0001	< 0.0001
*Bacteroides*	0.2 ± 0.044	26.7 ± 0.442	0.2 ± 0.044	1.4 ± 0.117	< 0.0001	NS	< 0.0001
*Prevotella*	0.0 ± 0.00	0.5 ± 0.070	0.1 ± 0.031	0.3 ± 0.054	< 0.0001	< 0.0001	< 0.0001
*Unc S24-7*	4.1 ± 0.115	4.9 ± 0.215	0.3 ± 0.054	1.4 ± 0.117	< 0.0001	< 0.0001	< 0.0001
*Unc C111*	0.7 ± 0.083	1.0 ± 0.099	0.4 ± 0.063	0.7 ± 0.083	0.0002	< 0.0001	< 0.0001
*Unc ACK-M1*	0.8 ± 0.089	1.2 ± 0.108	0.5 ± 0.070	0.7 ± 0.083	< 0.0001	< 0.0001	< 0.0001
*Rothia*	0.0 ± 0.000	0.0 ± 0.000	0.7 ± 0.083	0.6 ± 0.077	NS	< 0.0001	NS
*Unc Coriobacteriaceae*	0.1 ± 0.031	0.0 ± 0.000	2.7 ± 0.162	0.1 ± 0.031	< 0.0001	< 0.0001	< 0.0001
*Adlercreutzia*	1.3 ± 0.113	0.1 ± 0.031	3.9 ± 0.193	5.6 ± 0.229	< 0.0001	< 0.0001	< 0.0001
*Atopobium*	0.0 ± 0.000	0.0 ± 0.000	0.0 ± 0.000	0.1 ± 0.031	NS	NS	< 0.0001
*Akkermansia*	0.0 ± 0.000	0.10 ± 0.031	0.0 ± 0.000	0.1 ± 0.031	< 0.0001	NS	< 0.0001

Values are expressed as the mean ± SD (n = 6). Data were analyzed by One-way ANOVA followed by Bonferroni post-hoc test, *p* ≤ 0.05, *NS=* not significant, CD: Standard- chow diet for 3 weeks; CD + A: Standard- chow diet for 3 weeks and subjected to amoxicillin treatment (50 mg/kg body weight) in 3rd  week; HFD: high-fat diet for 3 weeks (73% energy from fat); HFD + A: High-fat diet for 3 weeks and subjected to amoxicillin treatment (50 mg/kg body weight) in 3rd week. Unc=Unculturable

**Table 7:** Effect of diet on mice gut microbiota at species level

Species	Relative abundance (%)				Significance (adjusted *p* value)		
	CD	CD + A	HFD	HFD + A	CD vs CD + A	CD vs HFD	HFD vs HFD + A
*Enterococcus casseliflavus*	0.0 ± 0.000	0.0 ± 0.000	0.0 ± 0.000	2.6 ± 0.159	NS	NS	< 0.0001
*Lactobacillus reuteri*	0.2 ± 0.044	0.0 ± 0.000	0.0 ± 0.000	0.0 ± 0.000	< 0.0001	< 0.0001	NS
*Blautia producta*	0.1 ± 0.039	0.0 ± 0.000	0.1 ± 0.039	1.8 ± .0.132	< 0.0001	NS	< 0.0001
*[Ruminococcus] gnavus*	1.1 ± 0.104	0.1 ± 0.039	1.9 ± 0.136	0.1 ± 0.039	< 0.0001	< 0.0001	< 0.0001
*Veillonella dispar*	0.0 ± 0.000	0.0 ± 0.000	0.1 ± 0.039	0.4 ± 0.063	NS	0.0031	< 0.0001
*Eubacterium dolichum*	0.1 ± 0.039	0.0 ± 0.000	0.2 ± 0.044	16.6 ± 0.372	< 0.0001	NS	< 0.0001
*Brevundimonas diminuta*	0.0 ± 0.000	0.0 ± 0.000	0.1 ± 0.039	0.1 ± 0.039	NS	0.0002	NS
*Haemophilus Parainfluenzae*	0.0 ± 0.000	0.0 ± 0.000	0.3 ± 0.054	0.1 ± 0.039	NS	< 0.0001	0.0013
*Bacteroides acidifaciens*	0.1 ± 0.039	18.1 ± 0.385	0.1 ± 0.039	0.2 ± 0.044	< 0.0001	NS	0.0021
*Bacteroides uniformis*	0.0 ± 0.000	0.0 ± 0.000	0.0 ± 0.000	0.3 ± 0.054	NS	NS	< 0.0001
*Prevotella melaninogenica*	0.0 ± 0.000	0.0 ± 0.000	0.0 ± 0.000	0.3 ± 0.054	NS	NS	< 0.0001
*Rothia mucilaginosa*	0.0 ± 0.00	0.0 ± 0.00	0.7 ± 0.083	0.50 ± 0.070	NS	< 0.0001	0.0002
*Akkermansia muciniphila*	0.0 ± 0.00	0.1 ± 0.039	0.0 ± 0.00	0.10 ± 0.039	< 0.0001	NS	< 0.0001

Values are expressed as the mean ± SD (n = 6). Data were analyzed by One-way ANOVA followed by Bonferroni post-hoc test *p* ≤ 0.05, *NS=* not significant, CD: standard- chow diet for 3 weeks; CD + A: Standard- chow diet for 3 weeks and subjected to amoxicillin treatment (50 mg/kg body weight) in 3rd  week; HFD: high-fat diet for 3 weeks (73% energy from fat); HFD + A: High-fat diet for 3 weeks and subjected to amoxicillin treatment (50 mg/kg body weight) in 3rd week

*Lactobacillus* is one of the most predominant genus in patients with Type 2 diabetes and Zucker Diabetic Fatty (ZDF) rats, and plays an active  role in the development of chronic inflammation of diabetes and contributes to insulin resistance [20]. *Desulfovibrio* on the contrary is believed to contribute to the impairment of gut barrier and promotes insulin resistance [21, 22]**.** There is a positive correlation of *Oscillospira* with fasting serum insulin, and with a reduction in mRNA expression *of Zonula occludens-1* (ZO-1) that prevent leakage of solutes from gut [22, 23]. *Streptococcus* can ferment sugars to yield lactic acid linked to the development of  metabolic diseases [24]. The enrichment of *Granulicatella* is associated with obesity [25]. *Allobaculum* plays a crucial role in lipid deposition by repressing the *Lipoprotein lipase* (LPL) enzyme by expressing *Angiopoietin-related protein* (ANGPTL4) [26]. *Rothia* is known for its versatile metabolic capacities and provides growth substrates to Gram-negative non-mucus-degrading bacteria including *Pseudomonas aeruginosa* [27]*.* Amoxicillin treatment in HFD-fed mice showed a significant reduction of *Lactobacillus*, *Turicibacter*, *Desulfovibrio*, *Oscillospira*, *Allobaculum,* *Streptococcus*, *Granulicatella* (*p* < 0.0001) and a slight decrease of *Rothia* compared to the HFD-fed mice. The amoxicillin-treated CD-fed mice showed a significant decrease of *Lactobacillus* (*p* < 0.0001), *Oscillospira* (*p* < 0.0001) and *Desulfovibrio* (*p* < 0.0001), when compared to the CD-fed mice.

Interestingly, amoxicillin-treated HFD mice showed a significant increase in the relative abundance of *Blautia*, *Coprococcus*, *Sutterella*, *Bacteroides* and, *Akkermansia* as compared to mice receiving HFD, and CD diets. However, amoxicillin treatment in CD-fed mice caused a significant decrease in the relative abundance of *Blautia* and *Coprococcus* (*p* < 0.0001)*,* and a significant increase in *Akkermansia* (*p* < 0.0001) compared to the CD-fed mice. In our study, we also observed a substantial increase in *Bacteroides acidifaciens, Bacteroides uniformis*, and *Akkermansia muciniphila* (*p* < 0.0001) in amoxicillin- treated HFD-fed mice compared to HFD-fed mice only. Likewise, amoxicillin treatment in CD-fed mice also increased the abundance of *Bacteroides acidifaciens* and *Akkermansia muciniphila* (*p* < 0.0001) compared to the mice receiving only CD. Importantly, *Blautia *has a negative correlation with the fasting plasma glucose levels and haemoglobin A1C (HbA1c) levels [28]**.** Further, *Blautia* also plays an important role in repressing the colonization of pathogenic bacteria in the intestine [29]. Similarly, *Coprococcus* is shown to negatively correlate with hypertension, body mass index (BMI), and body fat percentage [30, 31]. *Bacteroides acidifaciens* and *Bacteroides unifor*mis induce *Glucagon-like peptide-1* (GLP-1) activation by using the *G-protein-coupled bile acid receptor* (TGR5) through bile acids, taurine, and cholate that counters obesity and increases insulin sensitivity in mice [32]. *Akkermansia muciniphila* decreases the expansion of inflammation markers in adipose tissue, lipid metabolism and improves insulin sensitivity [33].

In this study, we also observed a slight rise in *Enterococcus* and a significant increase in *Eubacterium, Klebsiella* and *Prevotella* (Table 6) in HFD-fed mice compared to the CD-fed mice (*p* < 0.0001)*. *Amoxicillin treatment of HFD-fed mice could not diminish this effect as substantiated by an abundance of *Enterococcus, Eubacterium*, *Klebsiella* and *Prevotella *(*p* < 0.0001) in these mice. Similarly, amoxicillin treatment in CD-fed mice also showed a substantial rise in *Klebsiella, Prevotella*, and *Helicobacter* compared to mice on CD only *(p* < 0.0001)*. *We also observed a considerable increase in *Enterococcus casseliflavus* and *Prevotella melaninogenica* in amoxicillin-treated HFD mice compared to mice receiving HFD alone, or CD alone (*p* < 0.0001).

Our results clearly indicate that treatment with amoxicillin reshapes the gut microbiota in both HFD-fed mice  as well as in CD-fed mice. Amoxicillin treatment could remodel the gut microbiota to allow inhabitation of beneficial microbes associated with improving the pathophysiology of metabolic syndrome as observed in HFD-fed mice. Nonetheless, amoxicillin treatment could not reduce the gut pathogens appearing in mice receiving HFD.

### Amoxicillin treatment in HFD-fed mice has non-significant impact on histopathology of vital organs

**Fig. 9:**
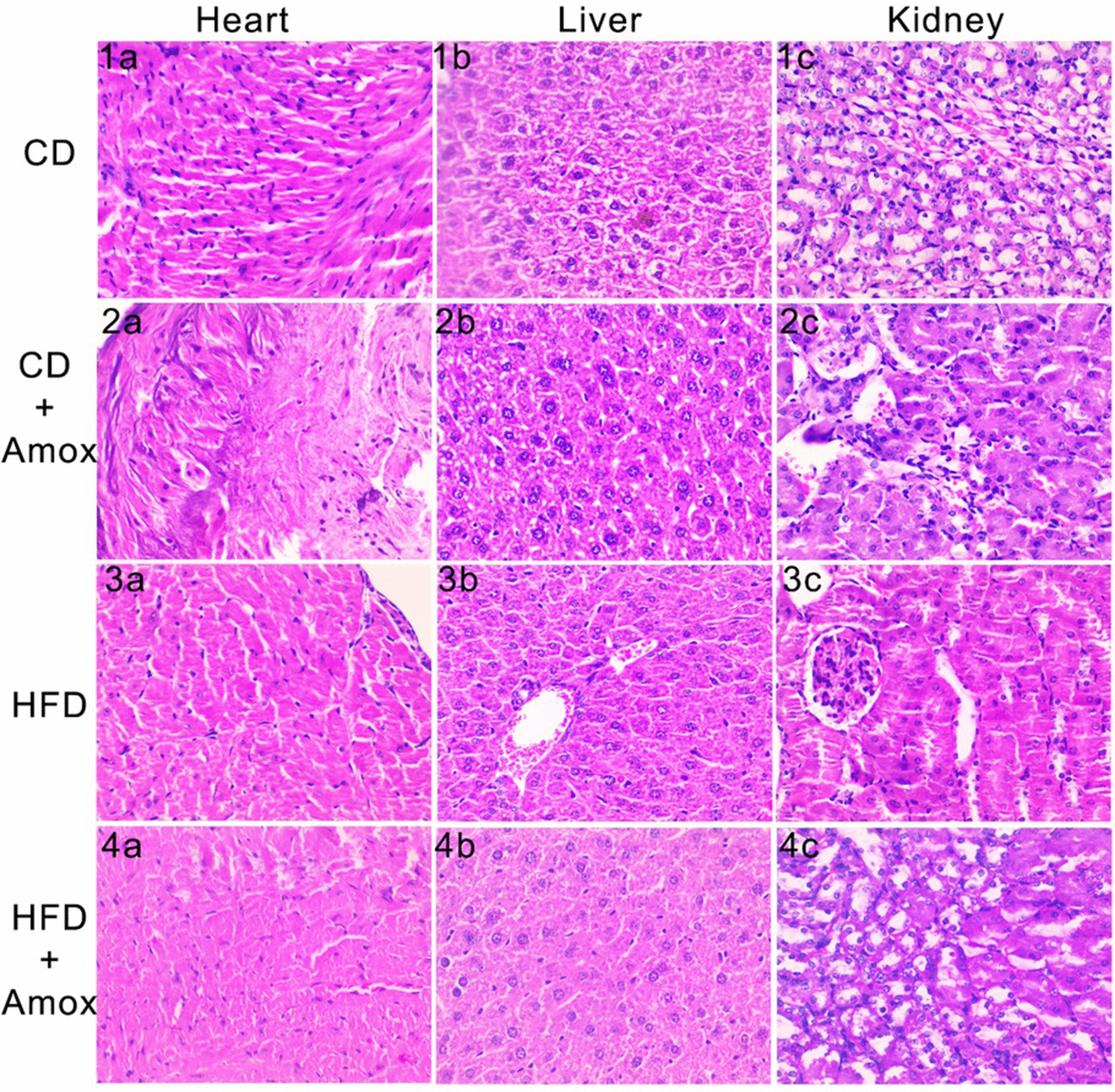
Impact of high-fat diet (3 weeks) and amoxicillin treatment on mice heart, liver, and kidney. The figure represents histopathogical examination of heart, liver and kidney sections by hematoxylin and eosin staining. The heart section of the HFD group showed congestion in the blood vessels (3**a**). Liver histology in the HFD group (3**b**) observed mild sinusoidal congestion of the central and portal vein. The kidney section in the HFD group showed normal architecture (3**c**). The heart (4**a**) liver (4**b**) and kidney (4**c**) histopathological in HFD + Amox showed no sign of morphological changes. CD: Standard- chow diet for 3 weeks; CD + Amox: Standard- chow diet for 3 weeks and subjected to amoxicillin treatment in 3rd week; HFD: High-fat diet for 3 weeks; HFD + Amox: High-fat diet for 3 weeks and subjected to amoxicillin treatment in 3rd week

To investigate whether antibiotic treatment could negatively impact the vital organs, we performed histopathological analysis of the heart, liver and kidney. We observed that no major histopathological damage occurs in CD-fed mice, HFD-fed mice and mice receiving amoxicillin treatment with these diets (Fig. 9). The heart section of HFD-fed mice exhibited mild congestion of blood vessels (Fig. 3a). Likewise, liver histology also indicated mild congestions in the sinusoidal, central and  portal veins in the HFD-fed mice (Fig. 3b). Amoxicillin treatment of these HFD-fed mice showed normal histopathological architecture of the heart, liver and kidneys (Fig. 4a–c). We did not observe any changes in histopathological analysis of CD-fed mice with or without amoxicillin treatment. Overall, albeit promising, these observations are insufficient to conclude a positive effect of amoxicillin treatment on improvement of mild histopathological changes observed in HFD-fed mice.

### Amoxicillin treatment in HFD-fed mice does not negatively impact macrophage's ability to clear the infection

**Fig. 10:**
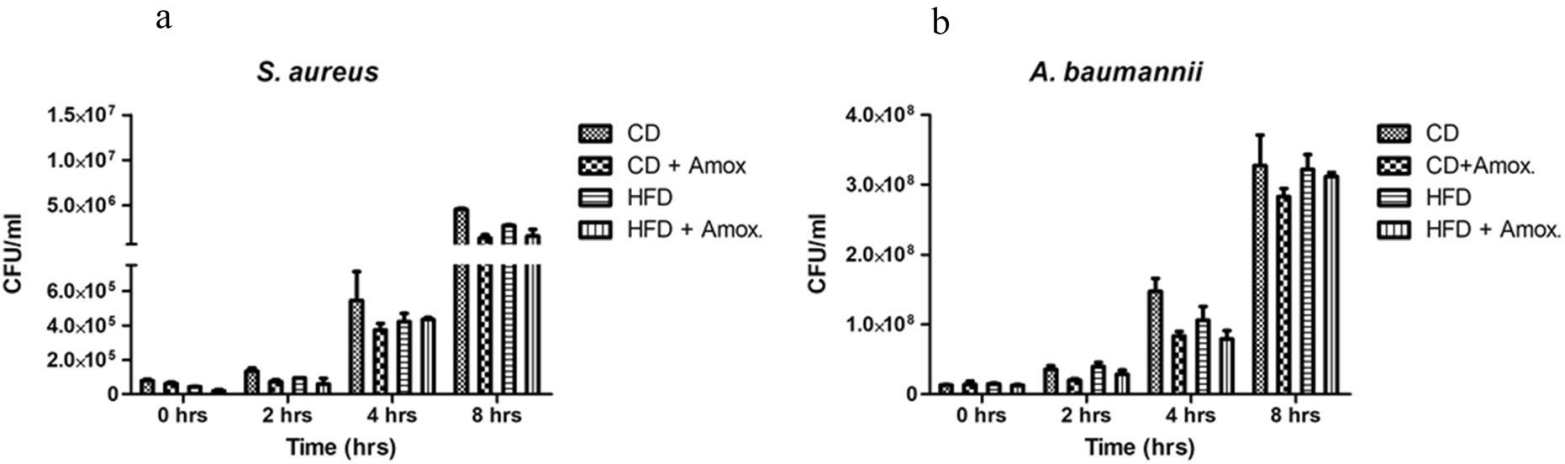
Impact of high-fat diet (3 weeks) and amoxicillin treatment on anti-bacterial activity of macrophages against *S. aureus* and *A. baumannii.* CFU recovery from peritoneal macrophages isolated from mice fed on different diets: Data represents mean ± SEM, One-way ANOVA followed by Bonferroni post-hoc test. *p* ≤ 0.05, NS=  not significant. CFU recovery of (**a**) *S*. *aureus*, and (b) *A. baumannii* at times 0, 2, 4, and 8 h (hrs) post-infection. There is no significant difference in CFU across the groups indicating that amoxicillin treatment does not significantly impair cell’s susceptibility and ability to clear microbial infections. All experiments were performed in triplicates. CD: Standard-chow diet for 3 weeks; CD + Amox: Standard-chow diet for 3 weeks and subjected to amoxicillin treatment  in 3rd week; HFD: High-fat diet for 3 weeks; HFD + Amox: High-fat diet for 3 weeks and subjected to amoxicillin treatment in 3rd week

We next wanted to investigate whether treatment with amoxicillin could impact the immune cell function associated with the anti-microbial action. Therefore, we analyzed the effect of amoxicillin treatment on antibacterial activity of primary immune cells i.e., macrophages. For this, we used macrophage infection assays with *S. aureus*, and *A. baumannii,* the two major clinically relevant microbial pathogens (Fig. 10)*.* We did not observe any significant difference in the recovery of colony forming units (CFU) from peritoneal macrophages isolated from mice fed on CD, CD + Amox, HFD and HFD + Amox at times 0, 2, 4, and 8 h post-infection. These data clearly show that treatment with amoxicillin did not impair the cell's susceptibility and  ability to clear microbial infections.

## Discussion

Metabolic syndrome induced by HFD has become a major health problem that increases the risk of individuals to many other chronic conditions. Despite the increased propensity to  infections in individuals, the link between duration of HFD and antibiotic treatment, and its impact on functionality and diversity of the gut microbiome and features of metabolic syndrome is not well established. In this study, we wish to investigate how antibiotic treatment influences the gut microbiota and other pathophysiological parameters in mice fed on a high-fat diet for a short period. Therefore, we performed a short-term HFD feeding experiment of 3 weeks involving amoxicillin intervention in the 3rd  week in a mice model. Firstly, our study has provided a conclusive  evidence that HFD, even for a short period, could lead to the reshaping of gut microbiota at the genus and the phylum level that are associated with the development of pathophysiological features typical of metabolic syndrome [34]. Further, we also observed that amoxicillin treatment preferentially improves these pathophysiological attributes associated with metabolic state during consumption of HFD.  According to our findings, HFD-fed mice showed an increase in taxonomic diversity and richness, particularly an appearance of specific gut bacteria such as *Unc Gemellaceae, Granulicatella, Enterococcus, Streptococcus, Dorea, Turicibacter, Veillonella, Allobaculum, Bilophila, Prevotella,* and *Rothia*. Nonetheless, amoxicillin treatment in HFD-fed mice broadly depleted these specific species namely *Gemellaceae, Granulicatella, Streptococcus, Dorea, Turicibacter, Allobaculum,* and *Rothia*. Intrestingly though, amoxicillin treatment of HFD-fed mice resulted in the exclusive appearance of specific gut bacteria such as *Unc Clostridiaceae, Sutterella, Flexispira, Helicobacter, Atopobium *and *Akkermansia*. These data show that each dietary group in our study had a distinct gut microbiota composition. 

Consistently we observed a significant increase in the *F/B* ratio associated with pathophysiological features of the obesity- associated metabolic state along with an increase in the relative abundance of *Proteobacteria* and *Actinobacteria* in HFD-fed mice [35, 36]. On the other hand, amoxicillin treatment of these HFD-fed mice almost restores the *F/B* ratio to  normal, and  comparable to the CD-fed mice with a concomitant decline in *Proteobacteria* and *Actinobacteria*. We also observed a significant reduction in *Lachnospiraceae*, and *S24-7* in HFD-fed mice. The depletion of these families are positively associated with disruption of gut epithelial barrier integrity and metabolic endotoxemia [4, 15]. The reduction in the relative abundance of *Lachnospiraceae* and *S24-7* was previously known for long-term HFD. That a 3-weeks HFD is sufficient to reduce the abundance of *Lachnospiraceae*, and *S24-7* is remarkable. However, treatment with amoxicillin in HFD-fed mice increased the relative abundance of *Lachnospiraceae* and *S24-7*. Therefore, our results suggest that amoxicillin treatment can be beneficial to moderate the inflammatory state associated with metabolic syndrome rather than further aggravating the pathophysiology of metabolic state observed with other antibiotics [37–39].

At genus level, we observed an abundance of *Lactobacillus, Turicibacter*, *Desulfovibrio*, *Oscillospira*, *Allobaculum*, *Streptococcus*, *Granulicatella*, and *Rothia* in mice receiving HFD only for 3 weeks. This is consistent with the previous reports on the abundance of these gut bacteria in the long-term high-fat diet [40–43]. This shows that alteration in gut microbiota takes place expeditiously following the changes in dietary habits. Further, most of the above-mentioned gut microbes contribute significantly to the development of pathogenesis of metabolic syndrome and gut inflammatory diseases. The abundance of *Lactobacillus* and *Turicibacter* positively correlates with blood glucose levels [19]. Further, several reports have indicated that an abundance of *Lactobacillus* is critical for the prevalence of obesity and contributes to the development of chronic inflammation in diabetes [44–47] Moreover, *Lactobacillus* is involved in insulin resistance [48] and impacts enzymatic activity of bile salt hydrolase to disturb lipid and glucose metabolism and contributing to development of Type 2 diabetes [49].

Similarly, the increase of *Desulfovibrio* and *Oscillospira* is  associated with development of insulin resistance [21–23]. Likewise, enrichment of *Streptococcus, Granulicatella*, and *Allobaculm* is associated with metabolic diseases [24–26]. *Rothia* is known to provide growth substrates to Gram-negative non-mucus-degrading bacteria [27]. The most striking feature in HFD-fed mice was the diminution of *Lactobacillus reuteri* that prevents the growth of pathogens in the gut [18, 19]. On the contrary, administration of amoxicillin in HFD-fed mice caused a significant decrease in *Lactobacillus, Turicibacter, Desulfovibrio, Oscillospira, Streptococcus, Granulicatella.,* and a slight decrease in *Rothia*. Therefore, our data suggest that amoxicillin treatment could positively improve the metabolic syndrome pathology by improving the insulin signalling and maintaining intestinal barrier integrity by decreasing the abundance of microbiota linked to obese related metabolic disorders and gut inflammatory diseases.

Next, we did not observe any significant change in the abundance of *Blauti*, *Coprococcus*, *Sutterella*, *Bacteroides*, and *Akkermansia* in HFD-fed mice as is also reported for the long-term HFD studies [42, 43, 50–52]. Amoxicillin treatment in HFD-fed mice showed an increase in the relative abundance of *Blautia, Coprococcus, Sutterella, Bacteroides* and *Akkermansia*. The increase in the relative abundance of *Coprococcus*, *Blautia, Bacteroides acidifaciens, Bacteroides uniformis*, and *Akkermansia muciniphila* [28–33] was  positively correlated with insulin sensitivity and counter of obesity. We also detected *Blautia producta* in amoxicillin-treated HFD mice which is known to inhibit the growth of pathogens [53]. Taken together, we argue that amoxicillin treatment during HFD can positively influence metabolic syndrome pathophysiology by augmenting the beneficial gut bacteria.

Apart from the beneficial effects of modulating gut microbiota by amoxicillin in HFD-fed mice, we consistently observed an increase in the relative abundance of other gut bacteria such as *Erysipelotrichaceae* [54], *Enterobacteriaceae* [24], *Bacteroidaceae* [51], *Enterococcus* [21], *Eubacterium* [24], *Klebsiella* [6] and *Prevotella* [11] in HFD-fed mice. Amoxicillin treatment could not diminish their abundance. The potential flourish of *Erysipelotrichaceae* after broad-spectrum antibiotic treatment has also been observed in earlier long-term HFD studies [55]. However, we observed a decrease of *Erysipelotrichaceae* in amoxicillin-treated CD groups. *Enterobacteriaceae* and *Bacteroidaceae* families are symbionts helping in maintenance of gut health [51]. However, their abundance has also been observed during acute infective processes [16]. We also observed a significant increase in abundance of these families in the amoxicillin-treated CD group. The rise in *Eubacterium* is associated with critical virulence factors that cause gut inflammation and various human disease states [56–58]. *Klebsiella* and *Prevotella* strains also have pathogenic properties that promote inflammatory bowel disease or other inflammatory diseases [59, 60]. It is worth mentioning that we detected *Enterococcus casseliflavus* and *Prevotella melanogenic* enrichment which is linked with inflammatory diseases [61, 62]. Thus, we observed that amoxicillin treatment in HFD-fed mice increased few gut intestinal bacterial species including potential pathogens that could impact metabolic syndrome pathology.

Consistently, we observed a significant increase in total cholesterol in HFD-fed mice. Amoxicillin treatment caused a slight but non-significant increase in the cholesterol levels which could be  attributed to the increase in expression of  hepatic *HMG-CoA reductase* [63, 64]. Nonetheless, amoxicillin treatment in mice receiving standard CD had significantly reduced cholesterol levels. We noticed a significant decrease in triglycerides (TG) concentration in high-fat diet mice, as is also shown in other studies [65]. This could be attributed to the elevated insulin concentrations that could inhibit hepatic very-low-density lipoprotein (VLDL) secretions and stimulate adipose TG uptake thereby  contributing to reduced serum TG levels [65]. On the other hand, amoxicillin treatment in HFD-fed mice did not cause any significant effect on the TG levels but caused a marked decrease in the CD group**.** Further amoxicillin treatment in HFD-fed mice also restored levels of AST suggesting a beneficial role of amoxicillin in the improvement of liver function.

We next observed that HFD for a short period (> 3 weeks) significantly increased blood glucose levels [66]. We also observed an alteration of gut microbiota in HFD-fed mice that could be directly involved in developing peripheral insulin resistance [66]. It is worth mentioning that amoxicillin-treatment of HFD-fed mice significantly reduced the blood glucose levels indicating its hypoglycaemic effect. Amoxicillin may cause depletion of the microbiome, which in turn could alter glucose homeostasis to affect systemic glucose metabolism via shaping gut microbial communities and regulating gene expression programs in the intestine and liver [67, 68]. The improvement in glucose tolerance following administration of antibiotics has also been observed with vancomycin and could be attributed to the increased abundance of *Akkermansia muciniphila*. We also observed a significant increase in the relative abundance of *Lachnospiraceae*, *S24-7*, *Oscillospira*, and an increase in the relative abundance of *Coprococcus*, *Blautia, Bacteroides acidifaciens*, *Bacteroides uniformis* and *Akkermansia muciniphila,* primarily restricted to amoxicillin-treated HFD-fed mice. These specific microbiotas could be involved in the restoration of blood glucose levels in this group.

Based on the above discussion, we believe our findings have an excellent clinical potential  as amoxicillin treatment in metabolic syndrome pathophysiology  could improve host insulin signalling, leading to a decrease in blood glucose level. There could be a concern about negative effects on the gut microbiota, exerted by antibiotics including reduced species diversity and altered metabolic activity which may lead to antibiotic-associated diarrhoea and recurrent *Clostridioides difficile* infections [9, 69, 70]. However, increasing reports about the beneficial effects of antibiotic treatment are emerging as well. For example, a blood pressure-lowering effect in a patient with treatment-resistant hypertension is observed when treated with a combination of antibiotics [71]. Therefore, the therapeutic intervention focusing on modulating microbiota by antibiotics is an attractive concept. Studies have also shown that fasting blood glucose and HbA1c levels in mice positively correlates with *Lactobacillus* abundance [20]. In our study, the reduction in the *Lactobacillus* abundance may explain improved glucose levels in amoxicillin-treated HFD-fed mice. Similarly, we also observed a significant remodelling of the gut microbiota towards a predominantly beneficial skew,  probably contributing to the improved biochemical and physiological outcomes in amoxicillin-treated HFD group. Therefore, our study supports the potential beneficial role  of amoxicillin treatment in short-term high-fat diet especially in individuals with diabetes as shown by restoration of blood glucose levels in HFD-fed mice. Nonetheless, further research would be required to explore the detailed mechanisms involving amoxicillin-mediated effects on blood glucose levels.

Long-term HFD studies have shown a significant decrease in haematological parameters [72, 73]. In our study, we observed a slight decline in the value of Hb, RBC, MCH and a considerable reduction in HCT and MCV in HFD-fed mice. Amoxicillin treatment in HFD-fed mice and CD group did not manifest any significant alteration in haematological parameters. Prolonged exposure to  HFD (> 4 weeks) induces a depressive effect on thrombocytes in mice [74]. HFD consumption elevates *IL-6* and thrombopoietin (TPO) levels that induce platelet production [75]. Remarkably, we observed that amoxicillin treatment caused restoration of the thrombocytes in HFD-fed mice towards the normal range. In contrast, amoxicillin treatment in the CD group did not have any significant effect on the thrombocytes value. Our data suggest that amoxicillin treatment in metabolic pathologies such as HFD could potentially restore thrombocytes towards normal value. Further, we also observed that amoxicillin benefits other blood circulating parameters including restoring levels of liver enzyme AST. Therefore, the possibility of improving liver function and reducing the risk of clotting, stroke or heart attack by amoxicillin appears to be an attractive preposition in individuals consuming HFD.

A short-term HFD intake influences the liver's susceptibility to inflammatory stimuli through the induction of a pro-coagulation state in mice livers [76]. This study observed a mild congestion in the blood vessels of the liver and heart in HFD-fed mice. Consistently, we did not observe significant damage to the kidneys [77]. Amoxicillin-treated HFD-fed mice showed normal heart, liver and kidney architecture. Our data revealed no significant histopathological changes to vital organs including the heart, liver and kidney. It is therefore clear that amoxicillin treatment did not cause any damage to the vital organs.

Next, we  also examined the impact of HFD on body weight and food intake. Our results are consistent with the established observations which demonstrate that weight increase is gradual, and it becomes apparent only after 4 weeks of treatment. Typically, only after 16–20 weeks of a high-fat diet, the mice exhibit ~ 20–30% increase in body weight compared to CD-fed mice [78, 79]. Previous studies have shown that rodents of different age groups fed on a long-term HFD display more body weight gain as compared to CD-fed animals [80, 81]. Moreover, the rate of weight gain in adult animals were higher due to their slower metabolic rate as compared to younger animals. However, amoxicillin treatment in HFD-fed mice did not show any significant difference on weight gain. Therefore, in our study, the lack of any significant weight gain in HFD-fed mice could be due to short-duration of HFD and/ or use of young adult mice.

Lastly, we also examined whether amoxicillin treatment in mice may adversely impact the ability of immune cells to clear microbial infections. By using two priority pathogens commonly occurring in hospital patients, namely *S. aureus* and *A. baumannii,* we found that amoxicillin treatment administered for a short duration (1-week) did not negatively impact cells' anti-microbial activity. Infection of peritoneal macrophages, one of the major cell types responsible for anti-microbial action, did not reveal  a significant difference in the CFU recovery across various groups, neither with *S. aureus* nor with *A. baumannii*. There is no adverse impact on macrophages' ability to clear the infection, emphasising the beneficial aspects of amoxicillin in the pathophyiological metabolic state. Thus the minor increase observed in the abundance of gut pathogens in amoxicillin-treated groups could be well taken care of by macrophages and would not be a major concern especially when other gut microbiota involved in improving insulin sensitivity are significantly improved.

## Conclusions

In summary, our findings support the idea that amoxicillin treatment in the high-fat diet has potential beneficial impact on biochemical parameters, as indicated by our hematology and serum biochemistry investigations. Furthermore, the gut microbiota undergoes  a significant remodelling towards a predominantly beneficial effect, probably contributing to the improved biochemical and physiological outcomes. In addition, there is no adverse impact on macrophages' ability to clear the infection, emphasising the beneficial aspects of an amoxicillin-based regimen for improved health outcomes. Therefore, amoxicillin may reduce the risk of heart attack, stroke or a clot in blood vessels, and promises to be an attractive proposition in individuals consuming HFD. Extrapolation for these findings from animals to humans however will require further research and more advanced studies.

Taken together, we suggest that amoxicillin has a beneficial effect on the pathophysiology of metabolic state during intake of HFD. We believe our study provides a proof-of-principle to support a prophylactic amoxicillin treatment in individuals having a high-fat diet pathophysiology for  better biochemical and gut health. Further studies would be required to evaluate long-term effects of antibiotic administration, especially in the context of diabetics.

## Methods

### Animal Model

According to the NIH guide for the Care and Use of Laboratory Animals (National Research Council, USA), all the experimental procedures were conducted and approved by the Institutional Animal Ethics Committee (IAEC) of All India Institute of Medical Sciences (AIIMS), New Delhi. All mice were maintained at temperature (22 ± 3 °C) and humidity (40–60%) on a 12 h light/dark cycle with free access to drinking water and rodent diet throughout the study. From the same cohort, twenty-four male C57BL/6 J mice of 6 weeks old (initial weight 16–18 g) were allocated into four groups (N = 6 mice/group). The standard chow diet (CD) (10% energy from fat) and high-fat diet (HFD, 73% energy from fat including lard, cholesterol, and veg oil) were fed to CD and HFD groups, respectively for 3 weeks. The formula for the HFD was as follows: lard (380 g /kg), cholesterol (10 g/kg), vegetable oil (60 g/kg), casein 75% (240 g/kg), vitamin mix (10 g/kg), cellulose (50 g/kg), mineral mix (5 g/kg), and starch (200 g/kg). The diet groups were assigned as follows: CD for control, HFD-treated, CD + Amox and HFD + Amox. The amoxicillin treatment was given in the 3rd week at 50 mg/kg body weight, i.e., 0.25 mg/ml in drinking water as per the recommended doses used with mice [82]. The treatment/diet group was CD + Amox for positive control, and HFD + Amox for treated mice. Body weight, water and feed intake was  recorded for 3 weeks. After 3 weeks, all animals were euthanized, and samples were collected to observe the effects of amoxicillin on HFD and CD groups.

### Blood, Tissue and Caecal samples Collection

After 3 weeks, mice from these groups were allowed to fast overnight, and the blood samples were collected via cardiac puncture as a terminal procedure. Following blood collection, the organs (Heart, Liver and Kidney) were harvested  for histopathological examination. Caecal content samples were taken shortly after dissection and immediately frozen in liquid nitrogen and stored at − 80 °C until further use.

### Haematology and Serum Biochemistry

Serum biochemistry was performed using serum auto-analyzer Screen Master 3000, Tulip, Alto Santa Cruz, India, then quantifying Coral GPO-PAP kit (CORAL Clinical systems, Goa, India) using manufacturers’ instructions. The haematology analysis was carried out using an automated vet haematology counter (Melet Schloesing Laboratories, Guwahati, India) as per the manufacturer's instruction.

### Histopathology

After euthanasia, the liver, kidney and heart tissues were dissected and were fixed overnight in 10% neutral buffer formalin and embedded in paraffin blocks. For haematoxylin–eosin (H & E) staining, tissue sections of 4–5 μm thickness were collected on poly-L-lysine (Sigma) coated slides using a microtome and stained according to standard protocols. The digital images were taken by light microscopy (Olympus CX-29: Olympus Optical Co. Ltd, Tokyo, Japan) and camera (Magnus DC 10).

### 16S rRNA Gene Sequencing

Caecal samples were sent for next-generation sequencing and genus analysis (DNA Xperts Private Limited, India). According to the manufacturer's instructions, the total genomic DNA of gut microbiota was extracted using Qiagen DNA Stool Mini Kit. DNA concentration and integrity were assessed by fluorometer and agarose gel electrophoresis, respectively. The 16S rRNA gene amplicon sequencing was performed on the Illumina MiSeq according to protocols described by Illumina Miseq high-throughput sequencer guide [83]. The hypervariable region V3- V4 of the bacterial 16S rRNA gene was amplified by using the universal primer 515f, 5'-GTGCCAGCMGCCGCGGTAA-3'; and 806r, 5'-GGACTACHVGGGTWTCTAAT-3' on the Illumina MiSeq platform.

### Microbial Bioinformatics Analysis

The raw data were filtered to capture clean reads by eliminating the adapter pollution and low-quality sequences [84]. By using FLASH (Fast Length Adjustment of Short reads v 1.2.1), the high-quality paired-end reads were combined with tags with an average read length of 252 bp [85]. According to their unique barcode and primer sequences, the reads were assigned to each sample using Mothur software (V1.35.1, http://www.mothur.org).   The barcodes as well as primers were removed to get effective Clean Tags. The tags were then clustered as OTU (Operational Taxonomic Unit) with a 97% similarity threshold [86].

Taxonomic classification of the representative OTU was assigned by using three commonly used databases [Greengenes, SILVA and Ribosomal Database Project (RDP)]. Finally, an OTU table and a phylogenetic tree were generated for diversity analysis by  calculating  Chao1, observed species, Shannon indices and Simpson indices [87].

### Bacterial Strain and Culture Condition

This study used two opportunistic pathogens, namely *Staphylococcus aureus* (MTCC # 1430) and *Acinetobacter baumannii* (MTCC # 1425) to evaluate the impact of treatment on macrophage health and infection [88]. Briefly, *S. aureus* and *A. baumannii* were cultured on LB broth at 37 °C overnight under shaking at 180 rpm [89]. We determined the OD590 of the growing cultures and used this to calculate the multiplicity of infection (MOI) for the experiment.

### Microbial infections in Primary Macrophages

Mice peritoneal macrophages (PM) were isolated from the peritoneal cavity as mentioned in our previous studies [90, 91]. RBCs were removed from macrophages by using the RBC lysis buffer followed by washing with 1X PBS. PM were cultured and grown in RPMI 1640 supplemented with 10% fatal bovine serum and penicillin–streptomycin (100 U/mL penicillin, 100 µg/mL streptomycin) at 37 °C in CO_2_ incubator for 3 days. For infection assays, 5 × 10^4^ cells/well were seeded in a 24-well plate in complete media and cells were allowed to rest for 24 h. After 24 h, macrophages were washed and infected either with *S. aureus* or with *A. baumannii* for 2 h with an MOI of 1:3. Subsequently, cells were treated with media containing antibiotics (gentamicin, 200 µg/mL) for 1 hour to eliminate extracellular bacteria. This time post-treatment was considered as time zero. Infected cells were kept for 2, 4 and 8 h in complete media to monitor progress of infection. For CFU enumeration, cells were lysed with 0.025% SDS at required time points and cell lysates were serially diluted and plated on LB agar plates. Plates were incubated at 37 °C overnight followed by CFU enumeration.

### Statistical Analysis

GraphPad Prism version 9.2.0 (3.2.0) for Windows, Graph Pad Software, San Diego, California USA, http://www.graphpad.comwas used for the statistical analysis of experimental data. Values are expressed as the mean ± Standard Deviation (SD). Groups were compared by One-way analysis of variance (ANOVA) followed by the Bonferroni post-hoc test for comparisons between multiple groups with a value of *p* ≤ 0.05 as the cut-off for statistical significance.

## Data Availability

The Sequence Read Archive (SRA) submission datasets generated and/or analysed during the current study are submitted to SRA data repository of National Centre for Biotechnology, USA. Submission Id: SUB11233254; Bio Project ID: PRJNA821450. https://submit.ncbi.nlm.nih.gov/subs/bioproject/SUB11233254/overview.

## Abbreviations

CD: Standard chow diet
HFD: High-fat diet
CD + Amox: Standard chow diet + Amoxicillin
HFD + Amox: High-fat diet + Amoxicillin
Hb: Haemoglobin
RBC: Red blood cells
MCH: Mean corpuscular haemoglobin
HCT: Haematocrit
MCV: Mean corpuscular volume
WBC: White blood cells
ALT: *Alanine aminotransferase*
AST: *Aspartate aminotransferase*
TG: Triglycerides
LPS: Lipopolysaccharide
ZO-1: *Zonula occludens-1*
ANGPTL4: Angiopoietin-related protein
LPL: *Lipoprotein lipase*
SCFAs: Short-chain fatty acids
HbA1c: Haemoglobin A1C
VRE: Vancomycin-resistant enterococci
GLP-1: Glucagon-like peptide 1
TGR5 receptor: G-protein-coupled bile acid receptor 5
IBD: Inflammatory bowel disease
NEC: Necrotizing enterocolitis
CRC: Colorectal cancer
VLDL: Very low-density lipoprotein
HMG-CoA reductase: *3-hydroxy-3-methyl-glutaryl-coenzyme A*
OTUs: Operating taxonomic units
PCoA: Principal coordinates analysis
PM: Peritoneal macrophage
MOI: Multiplicity of infection
OD: Optical density
MTTC: Microbial type culture collection and gene bank
RPMI: Roswell park memorial institute
CFU: Colony forming unit
SDS: Sodium dodecyl sulphate
